# 

**Published:** 1912-12

**Authors:** 


### CONTENTS.

ORIGINAL COMMUNICATIONS:—

Nystagmus With Report of Some Rare Cases. By A. W. Stirling, M. D., M. B., C. M., (Edin.) D. P. H. (Eng.,) Atlanta, Ga. ---------- 455

Discussion of Dr. Claude Smith's Paper on "Diptheria, Its Diagnosis, Etc. ---------- 465

Clinical Society of the New York Polyclinic Medical School and Hospital ---------- 465

Epididymitis. By Ballenger & Elder, Atlanta, Ga. ---------- 469

EDITORIAL ---------- 473

SELECTIONS AND ABSTRACTS ---------- 476

BOOK REVIEWS ---------- 495

MEDICAL ITEMS ---------- 502

